# Over-Wrapping of the Aortic Wall with an Elastic Extra-Aortic Wrap Results in Luminal Creasing

**DOI:** 10.3390/jcdd5030042

**Published:** 2018-08-11

**Authors:** Christian Legerer, Zakaria A. Almsherqi, Craig S. McLachlan

**Affiliations:** 1 Rural Clinical School, Faculty of Medicine, University of New South Wales, Sydney, NSW 2052, Australia; z.almsherqi@unsw.edu.au (Z.A.A.); reperfusion@hotmail.com (C.S.M.); 2 Yong Loo Lin School of Medicine, National University of Singapore, Singapore 119228, Singapore

**Keywords:** aortic stiffness, elastic wrapping, aortic distensibility, single-layered aortic wall

## Abstract

Elastic extra-aortic wrapping is a potential non-pharmacological way to improve aortic compliance and treat isolated systolic hypertension associated with a stiffened aorta. We aimed to use computer simulations to re-evaluate whether there is aortic shape distortion in aortic wrapping to achieve greater elasticity of the wrapped aortic segment. Non-linear transient numerical analysis based on an idealized hyper-elastic single-layered aorta model was performed to simulate the force/displacement regimes of external aortic wrapping. Pressure-displacement relationships were used to establish model aortic wall distensibilities of 4.3 and 5.5 (10^−3^ mmHg^−1^). A physiological pulsatile lumen pressure was employed to estimate the potential improvements in aortic distensibility by compression forces representing elastic aortic wrapping. In the less distensible model of the aortic wall there was increased systolic expansion in the wrapped segment. We found a risk of creasing of the aortic luminal wall with wrapping. Sufficient unloading of a thick and elastic aortic wall to induce increased compliance, as observed in elastic wrapping, is associated with the potential risk of over compression and folding (creasing) inside the lumen.

## 1. Introduction

**Extra Aortic Wrapping.** Reduced aortic distensibility results in high pulse pressure, which is further augmented by wave reflection (pressure difference between the first and second systolic peaks) [1]. Augmentation is a component of both distensibility and peripheral resistance [2]. A reduction in aortic distensibility is a common pathology and also related to aging. Improving the distensibility of the aorta may be achieved by an elastic extra-aortic wrap. Such a wrapping of the external surface of the aorta reduces its diameter transiently during pulsatile flow. In experimental models such an extra-aortic wrap has been shown to reduce both systolic blood pressure and pulse pressure [3]. Additionally a multi-branched mathematical computer model [3,4] has been used to confirm these results.

Wrapping a stiffened aorta with a highly elastic material reduces the vessels cross-section area in diastole. If, in systole, the wrapped aorta expands further than the untreated vessel, pulse pressure will decrease. However, this is dependent on the stiffness ratio of the wrap material and the wrapped blood vessel. Such an elastic wrap may unload the vessel wall so that the pressure increase of the pulsatile circulation is absorbed by the wrapping material. Published studies have demonstrated the potential of the elastic aortic wrap to reduce arterial stiffness in an in vitro model of the aged human aorta and in a sheep aorta [3,4,5]. These studies suggest that wrapping of the ascending aorta is sufficient to reduce aortic pulse pressure by as much as 23%. However, Iliopoulos [3] also reports in vivo ovine experiments in which the aortic wrapping procedure was applied to the proximal descending thoracic aorta which showed no improvement in pulse pressure, and there was a notable increase in aortic stiffness. We suggest this is of little surprise as the investigated aorta was of a young sheep and, therefore, had a high compliance. To improve an already compliant circulation requires precise adjustment of the relation between arterial stiffness and the amount of compression with an elastic material. Specifically, the aims were to use computer simulations to re-evaluate whether there is aortic shape distortion in aortic wrapping to achieve greater elasticity of the wrapped aortic segment.

## 2. Methods

**Numerical modelling.** Non-linear transient simulations of a single-layered aorta model were performed in ANSYS Workbench 18.1 (Canonsburg, PA, USA). We employed a physiological pressure wave to an idealised representation of the ascending aorta. Firstly, reference distensibilities for different geometries and constant material properties were established. Subsequently, a cross-section of the aortic model was compressed to simulate the elastic aortic wrapping procedure. To minimise computational resources our structural-only model did not incorporate a fluid-structure-interaction analysis and, instead, combined longitudinal pre-stretch with a pulsatile pressure for a realistic representation.

**Figure 1:**
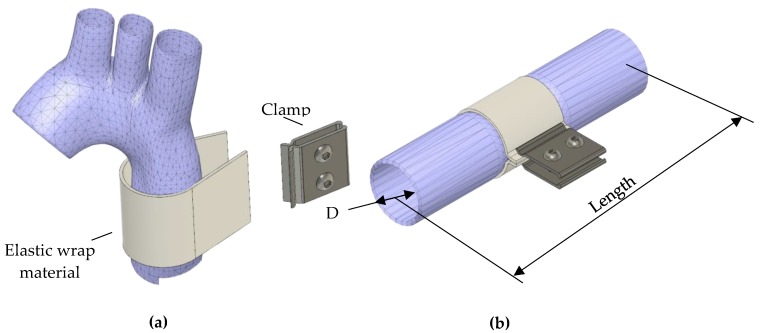
(**a**) Schematic representation of elastic aortic wrapping procedure on an idealised aorta, reproduced from [3]; and (**b**) the dimensions of the model aorta geometry (shortened for the purpose of illustration).

A simplified 3D model of the ascending aorta was developed using ANSYS SpaceClaim (Canonsburg). The idealized single layered tube geometry is depicted in Figure 1. Published dimensions of the ascending human aorta vary across published case reports [6,7,8,9]. We modelled a lumen diameter in the lower range, 20 mm (pre-expansion) [10]. Based on the summary of aortic thicknesses by Humphrey and G.A. Holzapfel [8], we used two aortic wall thicknesses, 1.5 and 2.5 mm. The age-dependent length of the ascending aorta is approximately 60–80 mm [11], to ensure comparability to other numerical studies and to eliminate boundary effects on the deformation behaviour the model tube was elongated to be 200 mm. The geometry was discretised with 3-D 20-node hexahedral solid elements that exhibit quadratic displacement behaviour and have mixed u-P formulation capabilities for simulating fully-incompressible hyper-elastic materials.

Mesh independence was established for maximum equivalent stress and peak von Mises stress in node located in the wall centre on the middle plane. Results for meshes with different numbers of circumferential elements are reported in Table 1. Von Mises, and equivalent, stress of Mesh 2 and Mesh 3 differed by less than 1%. Consequently, Mesh 2 with 30 circumferential elements was selected for our analysis due to the acceptable computational time and its consistency with the reported mesh in [12]. A more detailed discussion of the implantation of the incompressibility constraint in hyper-elastic constitutive models can be found in studies by Bucch and Hearn [13].

The following assumptions were made: that the aortic wall is a hyper-elastic, homogeneous, incompressible, and isotropic material. We applied a Yeoh constitutive model [14] with the strain energy potential, W which is a function of the first strain invariant, $\bar{I_{1}}$ and material constants, $c_{10},$ $c_{20},\;c_{30}:$

(1) $$W=c_{10}\;\left(\bar{I_{1}}-3\right)+c_{20}\;{\left(\bar{I_{1}}-3\right)}^{2}+c_{30}\;{\left(\bar{I_{1}}-3\right)}^{3}$$

Table 2 shows the material data published by Gelidi et al. [10]. Prior to the application of lumen pressure or compression, the aortic model was elongated in the axial direction by 10% of the initial length. This pre-stretch ensures numerical stability [15,16], without the use of un-physiological Rayleigh dampening [10]. Both longitudinal ends of the aortic model were constrained in the two remaining directions. Studies were performed to compare the modelled distensibility with literature and simulate the application of an elastic extra-aortic wrap.

**Distensibility.** The geometry/material properties simulated a numerical distensibility model comparable to published clinical data [17,18,19,20]. Transient structural analyses were run for 3 s with a 10% axial pre-stretch followed by a ramped pressure to the inner cylinder wall of 0.02 MPa/s. Radial displacement and stress/strain relationships were recorded to calculate the distensibility of the model aortic wall. Aortic distensibility is a parameter to describe the ability of the aortic wall to expand and contract and is defined as follows [17]:

(2) $$\mathrm{Aortic}\;\mathrm{distensibility}=\frac{Difference\;in\;cross\;section}{Diastolic\;cross\;section\cdot Pulse\;Pressure}\;[10^{-3}\;{\mathrm{mmHg}}^{-1}]$$

**Figure 2:**
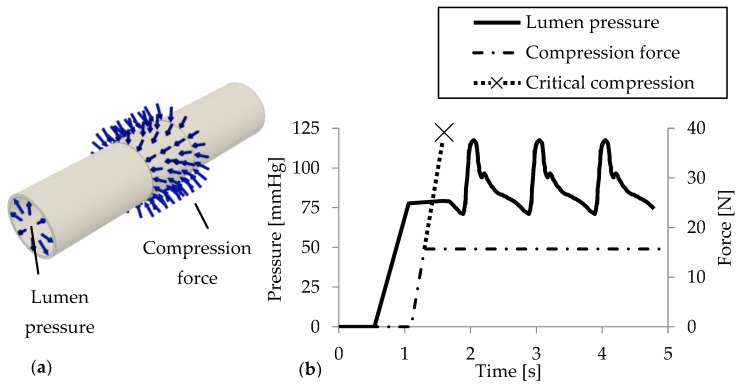
Aortic model with specified boundary conditions (**a**) and time-dependent lumen pressure load (**b**) [10].

**Extra aortic elastic wrapping procedure**. Figure 1 schematically depicts the application of an elastic wrap material to the ascending aorta as described in [3]. Figure 2a depicts the aortic model highlighting the luminal pressure and a 20 mm long segment with force vectors representing the simulated elastic wrap. Prior to compression, the aortic model was pre-stretched and gradually inflated to a luminal pressure of 75 mmHg (Figure 2b). The simulated surgical application of the elastic aortic wrap was performed by ramping the compression force to 16 N where it was held constant (dot-dashed line in Figure 2b). Physiological pulsatile pressure is superimposed. The force/displacement needed to achieve changes in functional compliance with extra-aortic wrapping was determined and stress responses determined using the finite element method.

Subsequently, in a second study the compression forces were sequentially increased to evaluate critical forces (dotted) and luminal surface changes. To reduce computational effort a symmetry boundary condition was applied to the transversal centre plan. Radial displacement was logged for representative deformation probes in the compressed sample centre and an unaffected area 60 mm from the centre.

## 3. Results and Discussion

The simulation outcomes are presented and interpreted in the following sections. First, distensibilities of the numerical models are correlated to in vivo values reported in the clinical literature.

**Figure 3:**
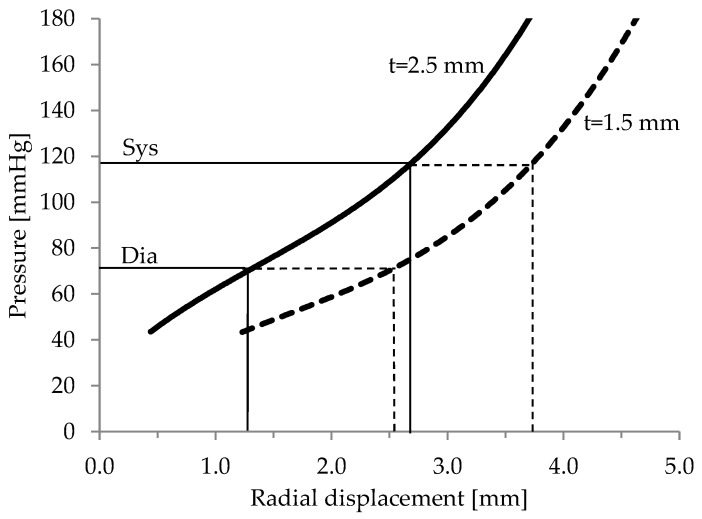
Radial displacement of node in aortic wall centre is plotted against the ramped inner wall pressure for t = 1.5 and t = 2.5 mm. Systolic (Sys) and diastolic pressure (Dia) of the pulse pressure wave are marked with horizontal lines.

**Reference distensibility.** The pressure-displacement relationship of the modelled aortic wall was established by plotting the applied lumen pressure against radial displacement. This curve demonstrated the calculation of aortic distensibility for physiological pressures according to Equation (1). For a normotensive pressure regime indicated in Figure 3, distensibilities of 4.3 and 5.5 are calculated for aortic wall thicknesses of 1.5 and 2.5 mm, respectively. Displacement values obtained with a linear ramped (increased) lumen pressure are identical to the displacement values of the unaffected area (proximal or distal to the wrapping) with pulsatile pressures.

The ascending aortic distensibility decreases from approximately 9 in children to 2 in old age [17,18,19,20]. Hence, our aortic model demonstrated a medium compliance, which (we suggest) should reduce the forces necessary to achieve a certain reduction in diameter.

**Extra aortic wrapping simulations.** A transient dynamic study was carried out to assess the feasibility of the elastic aortic wrapping procedure. A pulse pressure wave was applied to an aortic model with a 20 mm long wrapped section. In this numerical simulation the idealised force exerted by the wrap material was represented by a displacement independent, constant force. Figure 3 compares the pulsatile displacement response in an untreated and treated (placement aortic wrap) sections along aortic models with 1.5 and 2.5 mm wall thicknesses, respectively.

**Figure 4:**
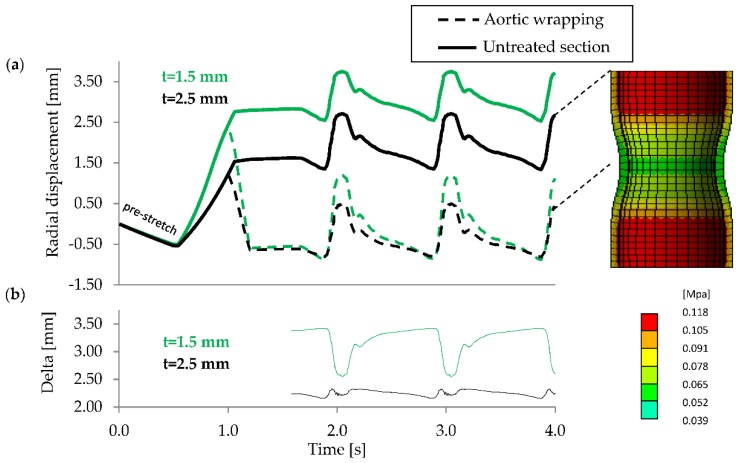
Radial displacement over time of two deformation probes along aortic model, comparing wall thicknesses of 1.5 mm and 2.5 mm (**a**); and the difference between untreated and compressed section for model thickness of 1.5 mm and 2.5 mm (**b**).

For the elastic wrapping procedure to improve the aortic compliance, the treated section needs to increase in cross-sectional diameter more than the untreated section during the systolic pressure rise. With an aortic wall thickness of 1.5 and 2.5 mm, respectively, the initial compression resulted in a 26% and 19% reduction in luminal diameter before pulsatile expansion. Following the initial compression in the two models (t = 1.5 and 2.5 mm), we noted that wall thicknesses influenced the responses, for example, they behaved differently regarding their displacement difference between untreated and treated segments of the simulated aorta (Figure 4b). As can be seen in Figure 4b, during systole the wrapped section of the aortic model with wall thickness 1.5 mm expands more than the untreated section, which results in a larger volume of blood to be accommodated, improving aortic compliance. On the contrary, compression of the 2.5 mm thick model resulted in an increased difference between treated and untreated segment, lowering the vessels compliance. In this case, the radial constriction would increase flow resistance.

As shown in the previous section, the initial inflation expands the 1.5 mm thick model further than the 2.5 mm model (Figure 3). This increases strain within the 1.5 mm thick wall, which leads to a lower distensibility (4.3) and a stiffer wall. In diastole the lumen diameter of the thinner model is about 2 mm wider. Similarly, a higher lumen pressure would increase the stiffness of the 2.5 mm model. Therefore, our results suggest that the effective application of an elastic aortic wrap depends on the aortic wall stiffness, thickness, diameter, and pressure. We propose that, in order for the elastic wrapping procedure to positively modify aortic distensibility and compliance of the more compliant, 2.5 mm thick model, the aortic wall needs to be unloaded. However, in our models this induces unfavourable creasing or folding of the wall.

**Figure 5:**
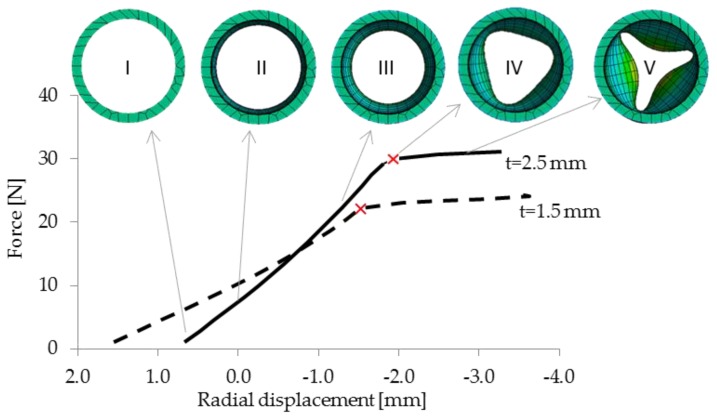
Force/displacement plot for ramped compression forces of elastic aortic wrapping in t = 1.5 mm and t = 2.5 mm. Critical forces which initiate folding are marked with red X. Pictures I-V above illustrate the progression of reduction in diameter with increased compression force for t = 2.5 mm.

**Formation of creasing in the aortic wall.** The extra aortic compression force applied to the central section of the aortic model was ramped until critical forces for folding of the model wall were reached (Figure 5). Initially, a gradual increase of the force, as expected in the surgical application of the elastic wrapping procedure, resulted in a linear reduction in diameter (I → III) (Figure 5). However, a critical force, which was evenly distributed over the aortic circumference, wrapped the aortic wall and caused it to fold (VI → V). In Figure 5 red Xs mark the inflection points, after which compression progressed more easily. This is accompanied by a significant reduction in the structural integrity of the aorta segment being wrapped. This can be seen and defined as ‘unloading’ of the aortic wall. At these reduced diameters a large proportion of the lumen pressure was transferred to the elastic wrapping material, possibly enabling improved expansion during systolic pulsatile flow. The maximal reduction in diameter before creasing and associated force are calculated to be 40% at 26 N and 30% at 43 N for wall thicknesses of 1.5 mm and 2.5 mm, respectively.

**Figure 6:**
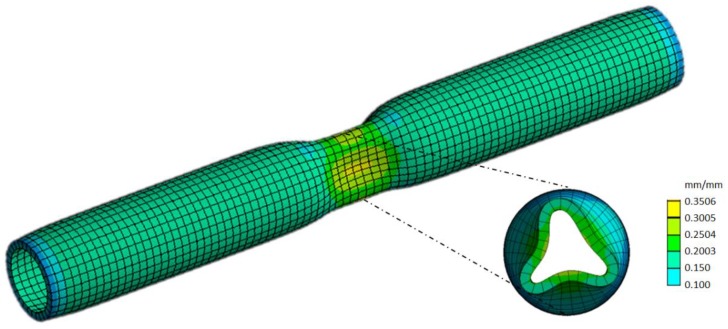
Exemplary representation of the strain regime in the aortic model, featuring a cross-sectional cut through the folding centre plane.

This simulation demonstrates that to successfully improve the compliance of an aorta with a distensibility of 5.5 and thickness 2.5 mm with an extra-aortic wrap, folding of the aortic wall is necessary for an effective load shift. We point out that this severe narrowing (Figure 6) in parts of the aorta increases the risk of platelet activation and concomitant blood clotting. Furthermore, turbulent flow caused by smaller radii could lead to further increased left ventricular afterload and vascular lumen shear stress. These pathological consequences would increase the risk of aortic dissection and embolic clotting.

Iliopoulos [3] reported the greatest reduction in pulse pressure, 23% for a reduction in diameter of 30% with the application of a silicone wrap with elasticity comparable to the young human aorta. Our results suggest that constrictions large than 30% or 40%(of the inflated diameter), for 2.5 mm and 1.5 mm wall thickness, respectively, lead to creasing of the aortic wall, which may well be the reason for the reduction in pulse pressure in these previous studies. However, narrow cross-sections associated with a folded aorta could lead to significant turbulences, risk of aortic dissection, and embolic clotting.

The results of this finite element study are limited to the applied strain-energy-function, selected material constants based on uniaxial experimental data reported in [10], and other numerical constraints. Furthermore, isotropy within a single-layered aortic wall is only an approximation of the blood vessels physiological behaviour. We believe that circumferential anisotropy would enhance the difficulty of uniform compression and risk of creasing. A multi-layered aorta with a less stiff adventitia would likely result in lower forces to achieve similar compressions and creasing. Therefore, we wish to emphasise that the main result of the presented work is to caution the clinician of the possibility of aortic creasing with aortic wrapping.

The shape of the folding aortic wall proposed is the result of numerical instabilities. Consequently, in-vivo this may differ considerably, depending on local wall anisotropy and, most importantly, non-uniformly distributed compression forces. To confirm our hypothesis additional computational efforts are required, which should incorporate anisotropy and effects of fibre, non-uniform geometries, different thicknesses, and a layered aortic wall. Ultimately, we want to determine the ideal amount of compression as a function of aortic stiffness, pulse pressure, diameter, and thickness.

## 4. Conclusions

The presented investigation shows that high compression forces in elastic extra-aortic wrapping could lead to folding of the aortic wall, which could increase turbulent blood flow and the risk for embolic events. We demonstrate that the effectivity of an extra-aortic wrap is dependent on the aortic stiffness, as a function of material properties, wall thickness, and diameter. For a compliant aortic model, low compression forces do not induce creasing, yet such compression is insufficient to unload the aortic wall. We report an improved compliance for the application of an elastic aortic wrap on the stiffer, thinner aortic model wall.

## Figures and Tables

**Table 1:** Maximum equivalent stress and peak von Mises stress of the centre probe at t = 1 s [MPa] on the aortic model during evaluation of critical compression forces.

	Mesh 1	Mesh 2	Mesh 3
Circumferential elements	15	30	60
Elements	1335	2670	5340
Nodes	9420	188,400	37,680
Max equivalent stress t = 1 s [MPa]	0.1015	0.1027	0.1030
Peak von Mises stress/centre probe t = 1 s [MPa]	0.0845	0.0934	0.0934

**Table 2:** Yeoh material constants for thoracic aortic tissue from uniaxial tensile testing by [10].

Yeoh Fitting Parameters	
C_10_ [Pa]	49,026.6
C_20_ [Pa]	−11,240.6
C_30_ [Pa]	63,666.5

## References

[1] O’Rourke M.F., Hashimoto J. (2007). Mechanical Factors in Arterial Aging: A Clinical Perspective. J. Am. Coll. Cardiol..

[2] London G.M., Pannier B. (2010). Arterial functions: How to interpret the complex physiology. Nephrol. Dial. Transpl..

[3] Iliopoulos J. (2006). The Aortic Wrap Procedure: A Surgical Method of Treating Age-Related Aortic Dilation and Stiffness.

[4] Giudici F., Qian Y., Rourke M.O., Avolio A. Simulation of reduction of proximal aortic stiffness by an elastic wrap and effects on pulse pressure. Proceedings of the 2012 Annual International Conference of the IEEE Engineering in Medicine and Biology Society.

[5] Avolio A.P., Butlin M., Protogerou A.D. (2014). Pulse pressure amplification and arterial stiffness in middle age. Blood Pressure and Arterial Wall Mechanics in Cardiovascular Diseases.

[6] Amabili M., Karazis K., Mongrain R., Païdoussis M.P., Cartier R. (2012). A three-layer model for buckling of a human aortic segment under specific flow-pressure conditions. Int. J. Numer. Methods Biomed. Eng..

[7] Mensel B., Kühn J.-P., Schneider T., Quadrat A., Hegenscheid K. (2013). Mean Thoracic Aortic Wall Thickness Determination by Cine MRI with Steady-State Free Precession: Validation with Dark Blood Imaging. Acad. Radiol..

[8] Humphrey J.D., Holzapfel G.A. (2012). Mechanics, Mechanobiology, and Modeling of Human Abdominal Aorta and Aneurysms. J. Biomech..

[9] Simsek F.G., Kwon Y.W. (2015). Investigation of material modeling in fluid–structure interaction analysis of an idealized three-layered abdominal aorta: Aneurysm initiation and fully developed aneurysms. J. Biol. Phys..

[10] de Gelidi S., Tozzi G., Bucchi A. (2017). The effect of thickness measurement on numerical arterial models. Mater. Sci. Eng. C.

[11] Krüger T., Forkavets O., Veseli K., Lausberg H., Vöhringer L., Schneider W., Bamberg F., Schlensak C. (2016). Ascending aortic elongation and the risk of dissection. Eur. J. Cardio-Thorac. Surg..

[12] Bucchi A., Hearn G.E. (2013). Predictions of aneurysm formation in distensible tubes: Part B—Application and comparison of alternative approaches. Int. J. Mech. Sci..

[13] Bucchi A., Hearn G.E. (2013). Predictions of aneurysm formation in distensible tubes: Part A—Theoretical background to alternative approaches. Int. J. Mech. Sci..

[14] Yeoh O.H. (1993). Some forms of the strain energy function for rubber. Rubber Chem. Technol..

[15] Rachev A. (2009). A Theoretical Study of Mechanical Stability of Arteries. J. Biomech. Eng..

[16] Badel P., Rohan C.P.-Y., Avril S. (2013). Finite Element simulation of buckling-induced vein tortuosity and influence of the wall constitutive properties. J. Mech. Behav. Biomed. Mater..

[17] Siegel E., Thai W.-E., Techasith T., Major G., Szymonifka J., Tawakol A., Nagurney J.T., Hoffmann U., Truong Q.A. (2013). Aortic Distensibility and its Relationship to Coronary and Thoracic Atherosclerosis Plaque and Morphology by MDCT: Insights from the ROMICAT Trial. Int. J. Cardiol..

[18] Güngör B., Yılmaz H., Ekmekçi A., Özcan K.S., Tijani M., Osmonov D., Karataş B., Taha Alper A., Mutluer F.O., Gürkan U. (2014). Aortic stiffness is increased in patients with premature coronary artery disease: A tissue Doppler imaging study. J. Cardiol..

[19] Voges I., Jerosch-Herold M., Hedderich J., Pardun E., Hart C., Gabbert D.D., Hansen J.H., Petko C., Kramer H.-H., Rickers C. (2012). Normal values of aortic dimensions, distensibility, and pulse wave velocity in children and young adults: A cross-sectional study. J. Cardiovasc. Magn. Reson..

[20] Groenink M., de Roos A., Mulder B.J.M., Spaan J.A.E., van der Wall E.E. (1998). Changes in aortic distensibility and pulse wave velocity assessed with magnetic resonance imaging following beta-blocker therapy in the marfan syndrome. Am. J. Cardiol..

